# Distal arthrogryposis syndrome

**DOI:** 10.4103/0971-6866.44108

**Published:** 2008

**Authors:** K. P. Kulkarni, I. Panigrahi, M. Ray, R. K. Marwaha

**Affiliations:** Department of Pediatrics, Advanced Pediatric Center, PGIMER, Chandigarh, India

**Keywords:** Contractures, distal arthrogryposis, whistling face

## Abstract

A 5-month-old male infant presented with weak cry, decreased body movements, tightness of whole body since birth, and one episode of generalized seizure on day 4 of life. He was born at term by elective caesarian section performed for breech presentation. The child had failure to thrive, contractures at elbow and knee joints, hypertonia, microcephaly, small mouth, retrognathia, and camptodactyly. There was global developmental delay. Abdominal examination revealed umbilical and bilateral inguinal hernia. Visual evoked response and brainstem evoked response audiometry were abnormal. Nerve conduction velocity was normal. Magnetic resonance imaging of brain revealed paucity of white matter in bilateral cerebral hemispheres with cerebellar and brain stem atrophy. The differential diagnoses considered in the index patient were distal arthrogryposis (DA) syndrome, cerebroculofacioskeletal syndrome, and Pena Shokier syndrome. The index patient most likely represents a variant of DA: Sheldon Hall syndrome.

## Introduction

Children with multiple joint contractures are frequently seen by practicing pediatricians and geneticists. It behooves them to be aware of the nosology, nature and management of these complex group of disorders collectively designated as arthrogryposis multiples congenita (AMC). We describe a case of distal arthrogryposis spectrum of disorders.

## Case

**Figure 1:**
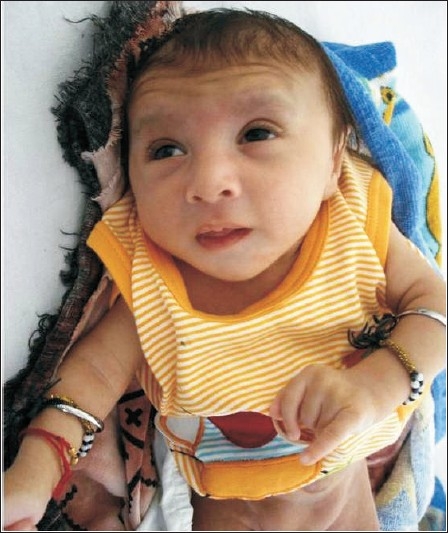
Facial features: microcephaly, deep set eyes, small mouth and retrognathia

**Figure 2:**
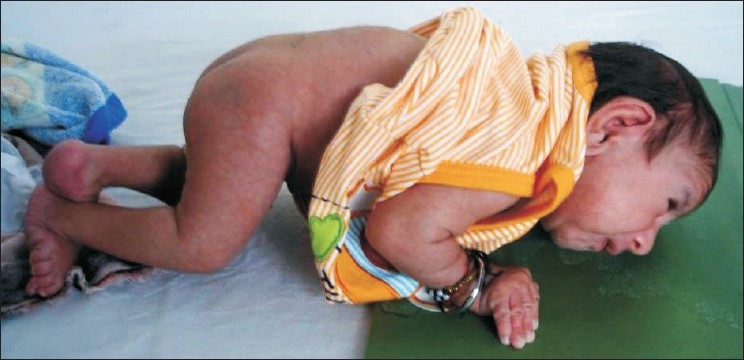
Full view: Contractures of elbow and knee, camptodactyly, ulnar deviation of hands, relatively long fingers, scoliosis, bilateral rocker bottom feet and Mongolian spots

**Figure 3:**
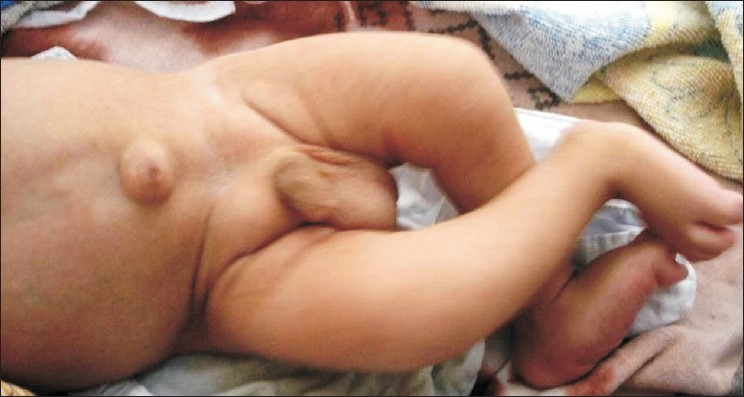
Umblical and inguinal hernia, scissoring of lower limbs and rocker bottom feet

A 5-month-old male infant presented with weak cry, decreased body movements, tightness of whole body since birth, and one episode of generalized seizure on day 4 of life. He was a product of nonconsanguineous marriage and was born at term by elective caesarian section performed due to breech presentation. The antenatal period was uncomplicated. There was no birth asphyxia or neonatal jaundice. The birth weight was 2.3 kg. The child was on exclusive breast-feeding. There was no history of previous abortions or stillbirths. There was family history of epilepsy in cousin who was otherwise developmentally normal. On examination, the child had weight of 3.8 kg (55% expected) and occipito-frontal circumference of 34.5 cm (<2SD). He had large ears, deep-set eyes, small mouth, retrognathia, camptodactyly, ulnar deviation of hands, relatively long fingers, mongolian spots, scoliosis, and bilateral rocker bottom feet [Figures 1-3]. There was no neck control, social smile, vocalization, visual fixation or following but the child was alert to loud sounds. He also had scissoring of lower limbs, contractures at elbow and knee joints, hypertonia, brisk deep tendon reflexes and up going planters [Figures 2 and 3]. Abdominal examination revealed umbilical and bilateral inguinal herniae but no hepatosplenomegaly [Figure 3]. The external genitalia were normal. The cardiovascular examination was within normal limits. There were no dysmorphic features in the parents or family members. The results of the salient investigations were as follows. The hemogram and the serum biochemistry were within normal limits. The karyotype was 46,XY. Ultrasound abdomen revealed no renal anomalies. Visual evoked potential (VEP) response showed delayed latency of P100 wave. Brainstem evoked response audiometry (BERA) showed delayed latencies of wave I, III and V. Magnetic resonance imaging (MRI) of brain revealed paucity of white matter in bilateral cerebral hemispheres, delayed myelination, thin corpus callosum, small volume posterior fossa, atrophy of cerebellum, and brainstem. Nerve conduction velocity was within normal limits.

Child was operated for inguinal hernia and physiotherapy was started. He was started on phenobarbitone for seizures and was reevaluated at 9 months of age when he persisted to have global developmental delay with no social smile, head holding or visual fixation. However, there was no recurrence of seizures.

## Discussion

AMC is non-progressive congenital syndrome complex characterized by contracture of several joints in different parts of the body. The genesis of AMC involves varying degrees of fibrosis of the affected muscles, thickening and shortening of periarticular capsular and ligamentous tissue with or without primary neurological or muscle disease that affect limb function.[1] Distal arthrogryposis (DA) is a clinically and genetically heterogeneous disorder characterized by clenched fist, overlapping fingers, camptodactyly, ulnar deviation, and positional foot deformities from birth.[2] Hall *et al*. classified these disorders into two main groups: DA type I (DA I), which is characterized by congenital contractures in two or more body areas without additional physical abnormalities, and DA type II (DA II), when other associated anomalies are observed as in the present case.[3] DA II is divided into five categories, named types A–E depending on the anomalies that are present in each patient. Bamshad *et al*. proposed an extended classification of DA with nine different types.[4] AMC is also a component of the fetal akinesia deformation sequence.[2] Pena Shokeir syndrome (PSS) and Cerebroculofacioskeletal (COFS) syndrome are prototype of the fetal akinesia spectrum of disorders.

With this background, the differential diagnoses considered in the index patient were (a) distal arthrogryposis syndromes: Freeman Sheldon syndrome (FSS, DAIIA), Sheldon Hall Syndrome (SHS, DAIIB) and (b) fetal akinesia syndromes: COFS and PSS. Major manifestations of FSS include ulnar deviation of the wrists and fingers, camptodactyly, hypoplastic, and/or absent flexion creases, and/or overriding fingers at birth, talipes equinovarus, and calcaneovalgus deformities, a vertical talus, and/or metatarsus varus with a characteristic small mouth, and whistling face appearance.[2,4] SHS shares features with DA1 and FSS.[2] Clinical features include a triangular face, downslanting palpebral fissures, attached earlobes, prominent nasolabial folds, small mouth, small mandible, arched palate, cervical webbing, short stature, severe camptodactyly, ulnar deviation, and vertical talus and/or talipes equinovarus. Patients with SHS do not have pinched mouth and H shaped dimpling of skin which are characteristic features of FSS. Additionally, as in the index patient, most patients with SHS do not have severe feeding difficulties after birth. In contrast, COFS is characterized by microcephaly, micrognathia, micropthalmos, neurodegeneration, contractures, and multiple skeletal malformations.[2,5] Thus, in view of the representative symptoms, clinical presentation and dysmorphic features; the index patient most likely represents a case of SHS. Moreover, PSS was a remote possibility due to low birth weight, large ears, kyphoscoliosis, and absence of pulmonary hypoplasia in the index case.[2,6] The findings on MRI in the index patient were in consonance with those reported by earlier investigators in DA syndromes. The delayed latency of P100 wave on VEP and those of waves I, III, and V on BERA can be explained by the paucity of white matter and hypomyelination in the index patient.

The commonest mode of inheritance in DA is autosomal dominant although autosomal recessive forms have also been reported.[7] Due to its solitary nature the mode of inheritance in the index case was unclear. The overlap of clinical characteristics of SHS with other DA suggests a shared etiology and/or pathogenesis. Authors have suggested role of mutations in TNN12 gene in the genesis of SHS.[8] Although prenatal diagnosis of DA depends upon ultrasound examination in late gestation, radiology and fetoscopy; recently, molecular prenatal diagnosis has been suggested for SHS.[9,10] Distinguishing among these disorders facilitates clinical management, directs anticipatory guidance, and increases the accuracy of recurrence risk estimates.
